# Metastatic retroperitoneal tumor from a non-functional neuroendocrine neoplasia of the left ethmoid-nose-orbitary region: Case report and short review of literature

**DOI:** 10.1016/j.ijscr.2019.12.001

**Published:** 2019-12-14

**Authors:** Carlo Cataldi, Saverio Cerasari, Gaetano Poillucci, Massimo Capaldi, Francesco Scocchera, Silvia Trombetta, Pietro Fransvea, Roberto Mazzarella-Farao, Pierluigi Marini

**Affiliations:** a General and Emergency Surgery, St. Camillo-Forlanini Hospital, Rome, Italy; b General and Emergency Surgery, St. Camillo-Forlanini Hospital, Rome, Italy; c Sapienza University of Rome, Italy; d General and Emergency Surgery, Giovanni Battista Grassi Hospital, Rome, Italy; e Emergency Surgery, Policlinico Universitario Agostino Gemelli Hospital, Catholic University, Rome, Italy

**Keywords:** NET, NeuroEndocrine Tumor, CT, computer tomografy, NSE, neuron specific enolase, DCG, dense core granules, APUD, amine precursor uptake decarboxylation, WHO, World Health Organization, NEN, NeuroEndocrine Neoplasm, HPF, high-power field, NEC, NeuroEndocrine Carcinoma, MEN, multiple endocrine neoplasia, MANEC, Mixed AdenoNeuroEndocrine Carcinoma, BINT, biologically inactive neuroendocrine tumor, MRI, magnetic resonance imagine, PET, positrone emission tomography, FDG, FluoroDeoxyGlucose, FLT, FLuoroThymidine, NETs, NENs, Metastatic neuroendocrine tumor, Retroperitoneal metastasis, Nose-orbital region

## Abstract

**Introduction:**

NeuroEndocrine Neoplasms (NENs) are rare and can originate from any epithelial organ. We describe a very rare case of retroperitoneal metastasis from a non-functioning neuroendocrine tumor of the ethmoid region and focus on the various issues related to NENs, from their nosological classification up to the most recent acquisitions in the diagnostic and therapeutic field.

**Presentation of case:**

A woman presented with a mass in the right retroperitoneal area that infiltrated liver and kidney. The anamnestic data showed a previous undifferentiated small cell tumor of the left ethmoid-nasal-orbital region. The mass was removed surgically and the definitive histological examination revealed a non-functioning undifferentiated malignant small cell neoplasm.

**Discussion:**

NENs represent a chapter of oncology whose systematization remains difficult. The lack of hormonal syndrome in Biologically Inactive Neuroendocrine Tumors (BINTs) may delay the diagnosis. Clinical manifestations relate to the size and location of the neoplasm. Small cell NeuroEndocrine Carcinoma (NEC) of the ethmoid-nose-orbital region is an extremely rare occurrence and generally the most frequent metastatic localization is represented by the larynx.

**Conclusion:**

This case highlights the contradictions typical of NENs and only new scientific acquisitions in the histopathological field can help us in the future. Surgery remains the only possibility of treatment and diagnosis of large masses of inactive NENs. This is the only case in the world of metastasis from NEC of the nose-orbital region in the retroperitoneum.

## Highlights

• NENs are rare tumors with extremely variable behavior: they are difficult to diagnose, especially if biologically inactive.
• NENs can originate from any epithelial organ and present unusual metastases.
• NEN surgery is a valid treatment and a necessary tool for correct diagnosis.
• NEN classification is evolving and being discussed.
• Careful collection of anamnestic data and a correct analysis of patients’ symptoms can be a valuable resource.

## 1 Introduction

NeuroEndocrine Neoplasms (NENs) are rare, accounting for 0.5 % of all new cancer cases [1]. They represent neoplasms that can originate from any epithelial organ, but most of them start from the gastrointestinal (70 %) and respiratory tracts (25 %) [2]. Today, the diagnosis of NEN is morphofunctional and is mainly entrusted to microscopic characteristics and immunohistochemical data in the presence of hormonal hypersecretion. In accordance with the World Health Organization (WHO) 2010 classification, we distinguished NeuroEndocrine Tumour (NET) G1 for well-differentiated endocrine tumors (including carcinoids), NET G2 for well-differentiated endocrine carcinomas, and NeuroEndocrine Carcinoma (NEC) for poorly differentiated endocrine carcinomas/small cell carcinomas [3]. The clinical case considered herein concerns the onset of a voluminous retroperitoneal metastasis from a non-functioning neuroendocrine primary neoplasm of the left ethmoid-sinus-orbital region treated exclusively with chemotherapy and radiotherapy cycles. The aim of our work is to focus on the various issues related to NENs, ranging from their nosological classification up to the most recent acquisitions in the diagnostic-therapeutic field, which condition the prognosis of these rare neoplasms. The work is described in line with SCARE criteria [4].

## 2 Case presentation

**Fig. 1:**
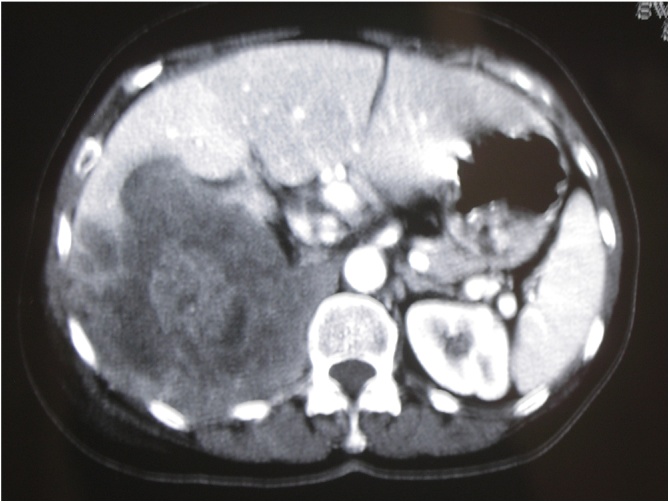
CT abdomen revealing a mass with a transverse diameter of 12 cm in the retroperitoneal region.

**Fig. 2:**
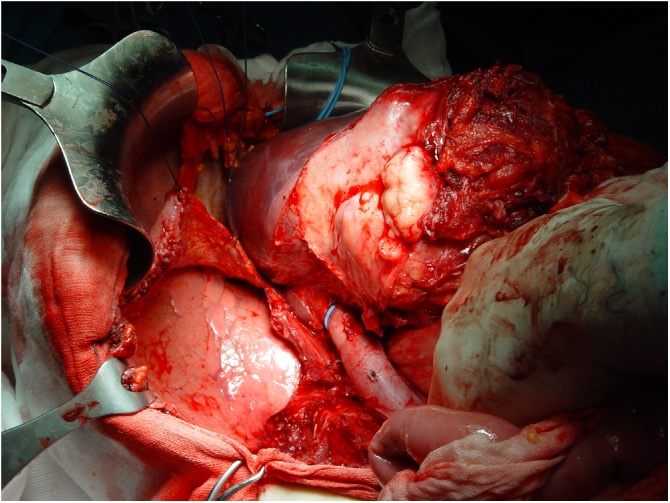
Removing kidney with perirenal fat.

At our institute, a 58-year-old female patient in good health came to our attention. Access to our department was determined by a persistent abdominal pain localized in the right lumbar region. The collection of anamnestic data revealed a small cell type NEC, discovered two years before, with positivity to Synaptophysin and Neuron Specific Enolase (NSE) but not to Chromogranin A, of the left ethmoid-nasal-orbital region, treated with chemo- and radiotherapy cycles. In our department the patient underwent a total body Computed Tomography (CT) scan (Fig. 1) which showed the presence of a voluminous mass in the right retroperitoneal area, with a maximum transverse diameter of 12 cm, presenting areas of necrotic colliquation in its context. This mass infiltrated the sixth and seventh hepatic segments, dislocated and infiltrated the inferior intrahepatic vena cava, dislocated the right kidney by infiltrating the superior pole and extended to the right lumbar region. This mass also infiltrated the anterior abdominal wall in the right lumbar region, determining an edema of the underlying subcutaneous adipose tissue, and dislocated, but did not infiltrate, the cephalic portion of the pancreas. Surgery was performed with total median laparotomic access. The mass was removed en bloc (Figs. 2, Fig. 33).

**Fig. 3:**
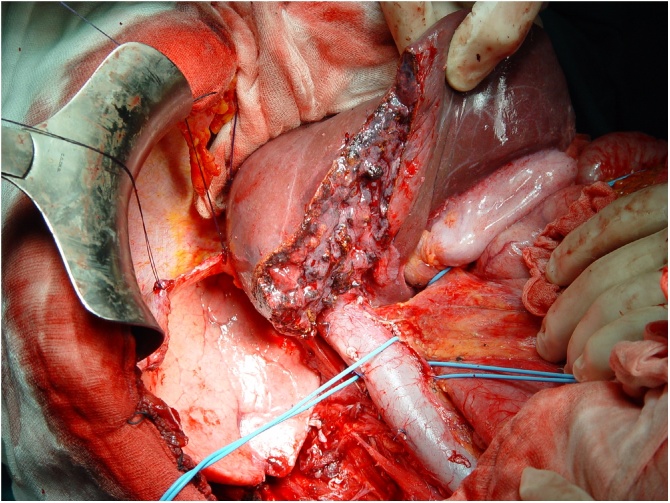
Removal of the entire mass with a part of VI and VII hepatic segment, right kidney with perirenal fat.

**Fig. 4:**
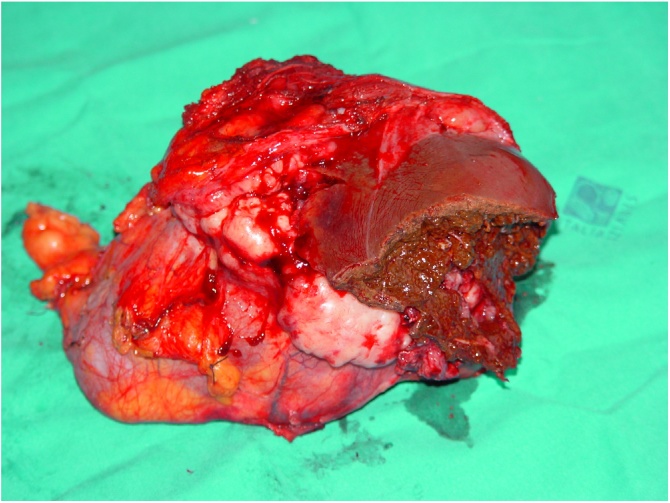
The entire mass surgically removed.

The whole mass weighed 1200 g, and was 17 × 13 × 12 cm in size, linked to all removed structures (Figs. 4, Fig. 55).

**Fig. 5:**
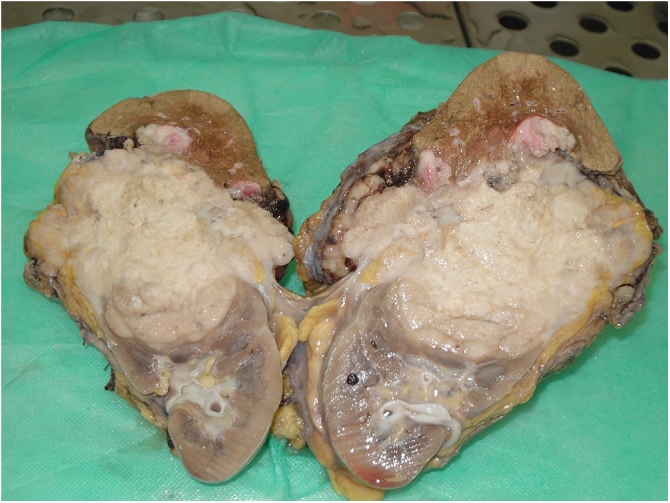
Cutting section of the neoplasm.

**Fig. 6:**
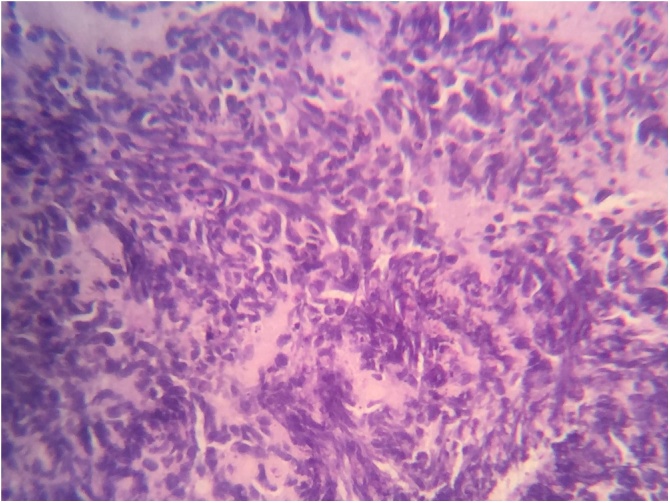
Definitive histological examination showed a small cell type NEC.

The final histological examination showed a NEC composed of small cells with immunohistochemical characters that were Synaptophysin-positive, NSE positive, but chromogranine negative (Fig. 6).

The post-operative course was satisfactory and the patient was released from the hospital on the 24th day in good condition and entrusted to oncologists for treatment of the case. The patient had a positive follow-up at 6 months and 12 months.

## 3 Discussion

The definition of neuro-endocrine cells has been changing over the years owing to improved knowledge and acquisition of modern research techniques. From the proposal put forward by Pearse in the 1960s and the introduction of the Amine Precursor Uptake Decarboxylation (APUD) system, which led to a common embryological histogenesis of these cells that showed their derivation from the neural crest, to the studies by Le Dourein, which highlighted that this was in part untrue, since the endocrine cells of the digestive tract, pancreas and lung are of endodermal origin [5]. NENs are rare neoplasms, representing about 0.5% of all newly diagnosed malignancies. Their distribution in general is: appendix 30%, colon-rectum 16%, stomach 14%, small intestine 9%, pancreas 6%, and medullary thyroid 5%. 20.8% of patients present with metastases [6]. NENs are a very heterogeneous group and originate from neuro-endocrine cells. They include carcinoids, non-carcinoid gastroenteropancreatic tumors, catacolamine-secreting tumors (pheochromocytomas, paragangliomas, gaglioneuromas, ganglioblastomas, sympathoblastoma, neuroblastoma), medullary thyroid carcinomas, pituitary tumors, small cell lung tumors and tumors of Merkel cells.

In 2010, WHO released a new classification of these neoplasms. Based on their mitotic rate (HPF, High-Power Field) and Ki-67 labeling index) [3] (Table 1) NENs were divided into:

1) NET G1 (mitotic count <2/10 HPF and/or ≤2% Ki-67 index);
2) NET G2 (mitotic count 2–20/10 HPF and/or 3–20% Ki-67 index);
3) NEC (mitotic count >20/10 HPF and/or >20% Ki-67 index);
4) Mixed AdenoNeuroEndocrine Carcinoma (MANEC), where at least 30% of either component must be identified to qualify for this definition;
5) Hyperplastic and preneoplastic lesions.

**Table 1:** Transition scheme for the new classifcation (WHO 2010) including previous definition for neuroendocrine neoplasms of the digestive system (WHO 1980 and 2000) [3].

WHO 1980	WHO 2000	WHO 2010
I. Carcinoid	1. Well-differentiated endocrine tumour (WDET)^a^ 2. Well-differentiated endocrine carcinoma (WDEC)^a^ 3. Poorly differentiated endocrine carcinoma/small cell carcinoma (PDEC)	1. NET G1 (carcinoid)^b^ 2. NET G2^b^ 3. NEC (large cell or small cell type)^b^, ^c^
II. Mucocarcinoid III. Mixed forms carcinoid-adenocarcinoma	4. Mixed exocrine-endocrine carcinoma(MEEC)	4. Mixed adenoneuroendocrine carcinoma (MANEC)
IV. Pseudotumour lesions	5. Tumour-like lesions (TLL)	5. Hyperplastic and paraneoplastic lesions

G, grade (for definition); NEC, neuroendocrine carcinoma; NET, neuroendocrine tumour.

a The difference between WDET and WDEC was defined according to staging features in the WHO 2000 classification. G2 NET does not necessarily translate into WDEC of the WHO 2000 classification.

b Definition in parentheses for the International Classification of Deseases for Oncology (ICD-O) coding.

c “NET G3” has been used for this category but is not advised, since NETs are by definition well-differentiated.

This classification is highly relevant to the prognosis and treatment of choice. The diagnosis of NEN is therefore morphofunctional and based essentially on microscopic features and immunohistochemical data: positive to Synaptophysin, to NSE and to Chromogranin. It is to be noted that well-differentiated NENs are intensely positive to the latter marker. This is true only in 10–15% of NENs in small cells are [7]. The study of genetic predisposition in the oncological diagnosis of NETs was possible thanks to the fundamental contribution of genetic analysis; these tumors occur in family groups carrying Multiple Endocrine Neoplasia (MEN) 1 and MEN2. These neoplasms are divided into Biologically Inactive Neuroendocrine Tumors (BINTs) forms and active forms. BINTs present clinical manifestations related to the size and location of the neoplasm. Signs and symptoms depend on the mechanical or mass effect of the tumor. A small cell NEC of the ethmoid-nose-orbital region is an extremely rare occurrence. It is divided into epithelial and neural, based on the presence of keratin or neurofilaments. Although rare, NECs and the olfactory neuroblastomas represent the most common neoplasia of the rhinosinusal tract [8]. The most frequent metastatic localization is the larynx, but other metastases can be found at the level of the cervical lymph nodes, lungs, liver, bone marrow and vertebrae [9]. Synaptophysin positivity also represents an extremely rare occurrence [10]. Less than 100 cases are reported in the literature and NEC is the most common in the ethmoid region [11]. It must be said that patients who have been treated with radiotherapy have an increased risk of developing secondary neuroendocrine cancers, and only two cases are reported in the literature [12]. The differential diagnosis with neuroblastoma, a tumor more easily found in this anatomical region, may present difficulties and only immunohistochemistry is decisive, even in controversial cases. These neoplasms have characteristics morphologically similar to those of small cell anaplastic tumors of lung cancer (Koss) [13]. The instrumental diagnosis makes use of ultrasound, depending on the primary lesion, biopsy samples, CT, CT angiography, Magnetic Resonance Imaging (MRI), immunoscintigraphy, Positrone Emission Tomography (PET), and a specific metabolic tracer (labeled 5-hydroxy tryptophan) which is able to selectively bind to NENs in 90% of cases showing lesions not visible on CT and MRI. The search for specific markers is very important. The therapy is multidisciplinary: surgery plays a fundamental role and must be as radical as possible, even using extensive demolitions, preceded when necessary by adjuvant and post-operative chemo- and radiotherapy. For the prognosis it is now established that the use of FluoroDeoxyGlucose (FDG)-PET is an excellent choice, with new research on 3′-deosoxy 3′-18F-FLuoroThymidine (18F-FLT), but it is not yet clear if it is useful for the choice of a specific treatment [14]. In the case that came to our attention, there was an interval that was free of disease, similar to the average survival in these cases, but in absence of surgery for removing the primary lesion. It is to be considered unusual that the resumption of the disease occurred through a voluminous retroperitoneal metastasis that required debulking, with sacrifice of the many organs involved, in absence of local recurrence of the primary tumor, thus representing the only case of retroperitoneal metastasis from NEC of the ethmoid-nose-orbital region described worldwide, although this region represents one of the most frequent locations of NECs of unknown origin [15].

## 4 Conclusion

NENs represent a chapter of oncology whose systematization remains difficult. Classification, study and research on these neoplasms entails complex knowledge involving all branches of medical science. The rarity of most NENs necessitates multicenter studies that allow histopathological classification. The non-active forms usually have a paucisintomatic symptomatology that is revealed only when the tumors occupy space. However, they are very aggressive neoplasms, especially in their poorly differentiated form. The diagnostic procedure includes ultrasonography, CT scan, immunoscintigraphy and PET. Immunohistochemical findings, and even those of genetic mutations, should not be overlooked for prognostic purposes. From a therapeutic point of view, surgery plays a fundamental role: it must be radical, with extensive lymphectomies and aggression of any metastases. The discovery of new biomarkers may open up new possibilities for classification, diagnosis and type of treatment. Ultimately, the clinical case presented herein highlights all the contradictions and problems typical of NETs and only new scientific acquisitions in the histopathological field will open new and more comforting scenarios in biological and therapeutic behavior.

## Sources of funding

This study did not receive any funding.

## Ethical approval

The study not require ethical approval.

## Consent

Written informed consent was obtained from the patient.

## Author contribution

Study concept or design: Saverio Cerasari, Carlo Cataldi

Data collection: Carlo Cataldi

Data interpretation: Saverio Cerasari, Carlo Cataldi

Literature review: Saverio Cerasari, Carlo Cataldi, Gaetano Poillucci

Drafting the paper: Saverio Cerasari

Editing of the paper: Saverio Cerasari, Gaetano Poillucci

Revision: Pierluigi Marini, Carlo Cataldi

## Registration of research studies

This study does not require registration.

## Guarantor

Carlo Cataldi

## Provenance and peer review

Not commissioned, externally peer-reviewed.

## Declaration of Competing Interest

The authors declare that they have no financial conflict of interest related to this paper.
